# Exopolysaccharides extracted from *Parachlorella kessleri* inhibit colon carcinoma growth in mice *via* stimulation of host antitumor immune responses

**DOI:** 10.1371/journal.pone.0175064

**Published:** 2017-04-05

**Authors:** Susumu Ishiguro, Deepthi Uppalapati, Zachary Goldsmith, Dana Robertson, Jacob Hodge, Hayley Holt, Arashi Nakashima, Katie Turner, Masaaki Tamura

**Affiliations:** Departments of Anatomy & Physiology, Kansas State University College of Veterinary Medicine, Manhattan, Kansas, United States of America; Duke University School of Medicine, UNITED STATES

## Abstract

The newly purified extracellular polysaccharides (exopolysaccharides) from *Parachlorella kessleri* (PCEPS) were evaluated on their antitumor and immunomodulatory effects in cell culture and mouse colon carcinoma peritoneal dissemination model. In two-dimensional cell culture, the PCEPS treatment inhibited cell growth of both murine and human colon carcinoma cells in a dose- and time-dependent manner. In contrast, the growth of mouse splenocytes (SPLs) and bone marrow cells (BMCs) were stimulated by the treatment with PCEPS. The treatment with PCEPS also increased specific subpopulations of the cells in BMCs: antigen presenting cells (CD19^+^ B cells, 33D1^+^ dendritic cells and CD68^+^ macrophage) and CD8^+^ cytotoxic T cells. In three-dimensional spheroid culture, spheroid growth of CT26 cells co-cultured with HL-60 human neutrophilic promyeloblasts and Jurkat cells (human lymphoblasts), but not THP-1 human monocyte/macrophage was significantly attenuated by PCEPS treatment. In a mouse CT26 colon carcinoma peritoneal dissemination model, intraperitoneal injection of PCEPS (10 mg/kg, twice per week) significantly attenuated the growth of CT26 colon carcinoma in syngeneic mice. The present study suggests that PCEPS inhibits colon carcinoma growth *via* direct cell growth inhibition and a stimulation of the host antitumor immune responses. Taken together, the current study suggests that exopolysaccharides derived from *Parachlorella kessleri* contain significant bioactive materials that inhibit colon carcinoma growth.

## Introduction

In the United States, colon cancer is the second cause of cancer death and there is an estimated at 95,270 new cases and 49,190 deaths in 2016 [1]. In the early stage of colon cancer, cancer is removed by polypectomy or local excision and good prognosis is reserved for the patient whose 5-year survival rate is 90%. However, survival rate declines to 70% and 13% for patients diagnosed with regional (lymph node) and distant (liver, lung and peritoneum) metastasis, respectively [2]. Although incidence and mortality of colon cancer have declined for the past decade because of development of effective early detections and treatments, this cancer contributes a significant portion of cancer-dependent mortality and morbidity.

*Chlorella* is a unicellular green algae and contains a variety of nutrients including amino acids, carbohydrates, vitamins, minerals and dietary fibers, therefore it is taken as a nutritional and functional dietary supplement worldwide [3]. In addition, it has been shown that whole dried powder and/or water extracts of *Chlorella vulgaris* and *C*. *pyrenoidosa* have therapeutic effects against several chronic diseases including hypertension [4, 5], hyperlipidemia [6, 7], viral infections [8, 9] and various cancers [10–16]. Although these studies identify that chlorella extract-dependent tumor growth inhibition is attributable to the stimulation of host antitumor immune responses [17, 18], the molecular mechanism by which chlorella extract stimulates immune responses is yet to be clear.

Recent studies also indicate that various microalgae produce a large amount of exopolysaccharides [19]. Exopolysaccharides are composed of a variety of polymeric carbohydrate molecules, such as alginate, cellulose, glucan, fucose, etc. and protect microbes from biotic and abiotic stress, such as interspecific competition, temperature, light intensity, pH, heavy metal stress, etc [20–23]. Although these exopolysaccharides produced by microalgae, such as cyanobacteria, are shown to exhibit apoptotic and antiviral activity in *in vitro* and *in vivo* [24, 25], bioactivity derived from chlorella/parachlorella has not been rigorously studied. Since these exopolysaccharides are presumably major components of whole chlorella water extract, it is of interest to study the biological activities in the field of cancer prevention and therapy. Here we report for the first time that exopolysaccharides derived from *Parachlorella kessleri* inhibit the growth of murine colon carcinoma cells *in vitro* in cell culture and *in vivo* in mouse allograft model *via* direct growth inhibition and stimulation of both neutrophilic promyeloblasts and lymphoblasts.

## Materials and methods

### Animals

Wild-type female Balb/c mice were obtained from Charles River Laboratories International, Inc. All mice were housed in a clean facility and held for 10 days to acclimatize. All experiments were carried out under the approvals of Kansas State University Institutional Animal Care and Use Committee (IACUC) and Institutional Biosafety Committee (IBC). All animal experiments were done under strict adherence to IACUC protocols set by Kansas State University (Manhattan, KS).

### Materials

Mouse colon carcinoma cell line CT26.CL25 (CRL-2639), human colon carcinoma cell lines SW620 (CCL-227), HT29 (HTB-38), COLO 205 (CCL-222) and Caco-2 (HTB-37), human neutrophilic promyeloblast cell line HL-60 (CCL-240), human monocyte/macrophage cell line THP-1 (TIB-202) and human lymphoblast cell line Jurkat (TIB-152) were purchased from American Type Culture Collection (ATCC, Manassas, VA). RPMI 1640 and Eagle's minimal essential medium (MEM) was obtained from Mediatech, Inc. (Manassas, VA). Macoy’s 5A modified medium was purchased from Sigma (St. Louis, MO). Fetal bovine serum (FBS) was from EQUITECH-BIO Inc. (Kerrville, TX). Penicillin-streptomycin stock was purchased from Lonza Rockland, Inc. (Allendale, NJ). Lipopolysaccharides from Escherichia coli O111:B4 was purchased from Sigma. Phycoerythrin (PE) conjugated antibodies targeting CD4 (H129.19), CD8b (YTS156.7.7), CD19 (6D5), dendritic cells (DCs) marker (33D1), LY6G (1A8), CD68 (FA-11) and mouse IgG1, κ isotype (MOPC-21) were purchased from BioLegend (San Diego, CA). OxPAPC (TLR2 and TLR4 inhibitor) and Polymyxin B (TLR4 inhibitor) were from InvitroGen (San Diego, CA).

### PCEPS preparation

Water extract from exopolysaccharides of *Parachlorella kessleri* (PCEPS) was obtained from PANAC ADVANCE Co., Ltd. (Tokyo, Japan). The PCEPS was prepared by the propriety method developed by the PANAC ADVANCE Co. Ltd. [26]. Briefly, culture media including *Parachlorella kessleri* were centrifuged at 6,500 x g for 5 min. Supernatant was concentrated by using an ultrafiltration membrane (SIP-1013, M.W. 6,000; Asahi Kasei Corp., Japan), then exopolysaccharides were purified by anion exchange resins. After re-concentration by the same ultrafiltration membrane, the sample was freeze-dried to obtain crude exopolysaccharides of *Parachlorella kessleri*. This crude extract was rehydrated at 4°C overnight. After centrifugation at 6,500 x g for 5 min, water soluble exopolysaccharides (PCEPS) containing supernatant was freeze-dried and subjected for the current study. Carbohydrate composition of PCEPS was analyzed by gas chromatography and found to be consisted of arabinose (4.7%), rhamnose (4.0%), xylose (1.7%), mannose (22.5%) and galactose (67.1%). The average molecular weight was determined by gel permeation chromatography and the major peak is shown to be approximately 65,000Da [26]. The PCEPS was dissolved in PBS at a stock concentration of 1 mg/ml, filtered through 0.22 μm disk filter (Midwest Scientific, Valley Park, MO) and the resultant filtrate was stored at -20°C until use. To ensure a negligible amount of lipopolysaccharide (LPS) contamination in this preparation, LPS levels in the final extract was determined by the Limulus amebocyte lysate (LAL) assay (Pierc LAL Chromogenic Endotoxin Quantitation Kit, Thermo Fisher Scientific, Waltham, MA). Assay results indicated that the final PCEPS preparation contained only very low levels of LPS or LPS-like immunoreactivity (average 0.1 ng per 1 μg PCEPS preparations, n = 3).

### Cell culture

The CT26 murine colon carcinoma cell line, SW620 and COLO 205 human colon carcinoma cell lines, HL-60, THP-1, and Jurkat cells were cultured with RPMI1640. The HT29 human colon carcinoma cells were cultured with Macoy’s 5A modified medium. Caco-2 human colon carcinoma cells were cultured with MEM. Each medium was supplemented with 10% v/v FBS and 1% v/v Penicillin-streptomycin. These cells were cultured at 37°C in a humidified air atmosphere containing 5% CO_2_.

### Effect of PCEPS on the growth of colon carcinoma cells, immune cells and mesothelial cells in two-dimensional (2D) cell culture

The murine (CT26) (1,000 cells/well) and human (SW620, HT29, COLO 205 and Caco-2) colon carcinoma cells (3,000 cells/well) were seeded into a 96 well plate with 100 μl growth medium. After 24 hrs, the cells were treated with PCEPS. The dose-dependent effect of PCEPS was evaluated by the cell growth treated with 1, 10 and 100 μg/ml PCEPS at 72 hrs after treatment. The time-dependent effect of PCEPS was evaluated by the cell growth treated with 100 μg/ml PCEPS at 24, 48 and 72 hrs after treatment. The cell proliferation was evaluated using 3-(4,5-Dimethylthiazol-2-yl)-2,5-diphenyltetrazolium bromide (MTT) assay as previously described [27]. PBS was used as the negative control. In addition, dose-dependency of PCEPS (1–100 μg/ml for 48 hrs) on the growth of immune cell lines was evaluated as described above by using human HL-60, THP-1 or Jurkat cells (1,000 cells/well).

The effect of PCEPS (24, 48 and 72 hrs treatment with 1–100 μg/ml PCEPS) on the growth of mouse normal mesothelial cells (3,000 cells/well) was evaluated by MTT assay as described above. Mouse mesothelial cells were collected from Balb/c mice as described previously [28] and cultured with RPMI-1640 medium supplemented 10% v/v FBS, 1% v/v penicillin-streptomycin, 10 μM 2-mercaptoethanol, 400 μg/L hydrocortisone and Insulin-Transferrin-Selenium-X (Thermo Fisher Scientific).

### Effect of lipopolysaccharides on the growth of colon carcinoma cells, immune cells in 2D cell culture

Lipopolysaccharides (LPS) are endotoxins found in the cell wall of Gram-negative bacteria and cyanobacteria [29, 30]. It is reported that Lipid A-like molecules, which are the phospholipid core of LPS, are immunologically detected in *Chlorella sp*. (strain NC64A) [31–33]. Therefore, it is conceivable that PCEPS may contain LPS-like molecules. In order to clarify the differences between PCEPS and LPS, the effect of LPS on the growth of colon carcinoma cell lines including murine CT26 cells (PCEPS sensitive), human HT29 cells (PCEPS sensitive), and immune cell lines (HL-60, THP-1 and Jurkat) was evaluated by MTT assay as described above.

### PCEPS activation of splenocytes and born marrow cells *in vitro*

The effect of PCEPS on the cell proliferation of splenocytes (SPLs) and bone marrow cells (BMCs) was evaluated by MTT assay in a 96-well plate. Balb/c mice were sacrificed by decapitation under anesthesia. SPLs were collected from spleen and cultured with RPMI-1640 supplemented 10% v/v FBS, 1% v/v penicillin-streptomycin, 20 μM 2-mercaptoethanol. BMCs were collected from born marrow of hind leg and cultured with RPMI-1640 supplemented 10% v/v FBS, 1% v/v penicillin-streptomycin. SPLs (5x10^5^ cells/well) and BMCs (2x10^5^ cells/well) were cultured in a 96-well plate. The cell proliferation was evaluated with dose-dependency (1, 10 and 100 μg/ml PCEPS for 48 hrs) and time-dependency (24, 48, 72 and 96 hrs treatment with 1 and 100 μg/ml PCEPS for SPLs, and 30 μg/ml PCEPS for BMCs). As a positive control, 100 ng/ml LPS was treated. PBS was used as the negative control.

### Characterization of TLR-mediated splenocyte stimulation using inhibitors

SPLs (5x10^5^ cells/well) were seeded into a 96-well plate, then treated with either 15 μg/ml OxPAC or 50 μg/ml Polymyxin B. After 30 min incubation, 10 μg/ml PCEPS or 100 ng/ml LPS were added. Cell proliferation was evaluated by MTT assay at 48 hrs after treatment. PBS was used as the negative control.

### Analysis of PCEPS-induced BMCs activation using flow cytometry

Born marrow cells (5x10^6^ cells) were seeded into a 6-well plate and incubated with 100 μg/ml PCEPS for 48 hrs. In order to evaluate percentage of immune cell population, cells were immunostained with anti-CD4 (helper T cells), CD8b (cytotoxic T cells), CD19 (B cells), DCs marker (33D1, dendritic cells), LY6G (neutrophil) and CD68 (macrophage) antibodies. Mouse IgG was used for the isotype control. The cell population was measured by flow cytometer (Moxi Flow; ORFLO, Ketchum, ID). PBS and LPS (100 ng/ml) were used as the negative and positive treatment control, respectively.

### Growth inhibitory effect of combination treatment by PCEPS and immune cells on the growth of CT26 spheroid in *in vitro* three-dimensional (3D) spheroid culture

In order to evaluate the combination effect by PCEPS and immune cells, a 3D spheroid assay was performed as described previously with slight modifications [34]. Briefly, five hundred of HL-60, THP-1 or Jurkat cells were mixed with 1.5% agarose dissolved in RPMI 1640 supplemented with 10% v/v FBS and 1% v/v penicillin-streptomycin. Fifty microliters of the mixture were added in a 96-well plate and the agarose layer solidified by standing at room temperature for 10 min. Then 1,000 of CT26 cells were seeded with growth medium on the agarose layer (Day 0). PCEPS (30 μg/ml) was treated twice at Day 1 and Day 4. The image of spheroids was taken by inverted microscope IX51 (Olympus America Inc., Center Valley, PA) equipped with cellSens Dimension software (Olympus) at Day 8 and 10. The spheroid growth was evaluated by spheroid area and was compared as a fold change to area at Day 8.

### Analysis of gene expression in immune cells co-cultured with PCEPS-treated CT26 cells in *in vitro* Transwell culture

The expression of CD11b, CD11c, and NADPH oxidase isoform (NOX2) in HL-60 and THP-1 cells, and TNFα, IFNγ, IL-2 and granzyme b (GZMB) in Jurkat cells co-culture with PCEPS-treated CT26 cells were evaluated by RT-qPCR. CT26 cells (1.5x10^4^ cells) and HL-60, THP-1 or Jurkat cells (5x10^4^ cells) were seeded in the Transwell inserts and corresponding 6-well plates, respectively (Day 0). After 24 hrs (Day 1), PCEPS (30 μg/ml) was added into Transwell inserts. Medium change and additional PCEPS treatment was carried out at Day 3 and Day 5. At Day 6, immune cells in the 6-well plates were collected and total RNA was purified using TRIzol reagent (InvitroGen). One step RT-qPCR was carried out using the iTaq Universal SYBR Green One-Step Kit (Bio-Rad, Hercules, CA), and the reactions were conducted on the StepOnePlus Real-Time PCR System (Applied Biosystems, Waltham, MA). The qPCR was performed as follows: 45 cycles of 15 seconds at 95°C, and 60 seconds at 60°C. The results were quantified by the comparative Ct method [35]. The sequences of primers used are described in Table 1.

**Table 1:** Primers used for RT-qPCR.

Primer		Sequence	Size
Human	Forward (5’-3’)	GCAACCTCTCGTTTGACTGG	149 bp
CD11b	Reverse (5’-3’)	CTCCACTTTGGTCTCCGTCTG	
Human	Forward (5’-3’)	CCTGTTGGCTTCTGTTCACC	157 bp
CD11c	Reverse (5’-3’)	GCTGTCGCCTTCTTTCTTCC	
Human	Forward (5’-3’)	AATGGTGTGTGAATGCCCGAG	222 bp
NOX2	Reverse (5’-3’)	GGATGGTTTTGGTGGAGGAAGTG	
Human	Forward (5’-3’)	GCCAGAATGCTGCAGGACTT	63 bp
TNFα	Reverse (5’-3’)	GGCCTAAGGTCCACTTGTGTCA	
Human	Forward (5’-3’)	AGGGAAGCGAAAAAGGAGTCA	64 bp
IFNγ	Reverse (5’-3’)	GGACAACCATTACTGGGATGCT	
Human	Forward (5’-3’)	ATGAGACAGCAACCATTGTAGAATTT	87 bp
IL-2	Reverse (5’-3’)	CACTTAATTATCAAGTCAGTGTTGAGATGA	
Human	Forward (5’-3’)	TGCAGGAAGATCGAAAGTGCG	180 bp
GZMB	Reverse (5’-3’)	GAGGCATGCCATTGTTTCGTC	
18S	Forward (5’-3’)	GAGGTTCGAAGACGATCAGA	315 bp
	Reverse (5’-3’)	TCGCTCCACCAACTAAGAAC	

### Treatment of tumor-bearing mice with intraperitoneal injection of PCEPS

The antitumor effect of PCEPS was evaluated in Balb/c mice with CT26 murine colon carcinoma allograft. Mice were anesthetized with isoflurane and intraperitoneally injected with 2.5x10^5^ CT26 cells suspended in 200 μl PBS. The intraperitoneal injection of PCEPS (10 mg/kg) was carried out at 1 week after CT26 inoculation for 11 days (every other day, total 6 injections). PBS was injected intraperitoneally as the control with the same schedule. The mouse body weights were monitored every other day. Three weeks after CT26 injection, all mice were sacrificed by decapitation under anesthesia and tumor nodules containing omenta and pancreases were collected to examine tumor growth. The tumor nodules containing two organs were weighed and fixed in 10% formalin for histological analysis. To evaluate the effect of PCEPS alone on organ weights, omenta (only normal mice), pancreases (only normal mice), lungs, livers, spleens and intestines were also collected from PBS- or PCEPS-treated normal or CT26 cell tumor-bearing mice and weighed. Since average weights of PBS- and PCEPS-treated mouse organs were not different at all, tumor nodule weights were normalized by subtracting the average weights of the omentum (425.2 mg ± 37.3 mg) and the pancreas (161.1 mg ± 27.2 mg).

### Flow cytometry analysis of immune cells in the ascites of PCEPS-treated tumor-bearing mice

CT26 cell tumor-bearing mice were prepared as described above (n = 4). Immune cells in ascites of PCEPS-treated tumor-bearing mice were collected at 2 days after the third treatment of PCEPS. Five milliliter of saline was injected into the abdominal cavity using 22Gx1¼” Surflash I.V. Catheter (Terumo Medical Corporation, Somerset, NJ) and ascites were collected *via* catheter. This ascites collection was repeated one additional time. After removing red blood cells using ACK lysing buffer (Lonza Walkersville, Inc., Walkersville, MD), leukocytes were immunostained using anti-CD4, CD8b, CD19, 33D1, LY6G and CD68 antibodies, and their population distributions were evaluated by flow cytometry. Mouse IgG was used for the isotype control. PBS was used as the negative treatment control. To evaluate the effect of PCEPS on the weights and appearances of various organs at midpoint of the PCEPS treatment (total 3 time treatments as compared to full scale treatment (total 6 time treatments)), tumor nodules containing omenta and pancreases, lungs, livers, spleens and intestines were dissected from PBS- or PCEPS-treated CT26 cell tumor-bearing mice, all organs were carefully observed and their weights determined.

### Analysis of PCEPS treatment-associated apoptosis of CT26 cell tumor cells by immunohistochemistry

TUNEL assay was carried out using the DeadEndTM colorimetric TUNEL system (Promega, Madison, WI) as previously described [36]. The average number of TUNEL positive cells in 10 random fields (n = 5–6) were calculated. Apoptotic cells in PCEPS-treated tumors were also evaluated using anti-cleaved caspase-3 antibody (1;300 dilution, for 18 hrs at 4°C, #9661, Cell Signaling Technology, Danvers, MA) as previously described [37]. The average number of anti-cleaved caspase-3 positive cells in 10 random fields (n = 5–6) was calculated.

### Statistical analysis

All values are expressed as the mean ± standard deviation of mean. For all *in vitro* and *in vivo* experiments, statistical significance was assessed by unpaired t-test or ANOVA followed by Tukey’s test. All experiments were conducted with multiple sample determinations with several samples (n = 3–5). Statistical significance was set at *, P<0.05.

## Results

### PCEPS treatment attenuated the growth of murine and human colon carcinoma cells, but stimulated human immune cells in 2D cell culture

**Fig 1:**
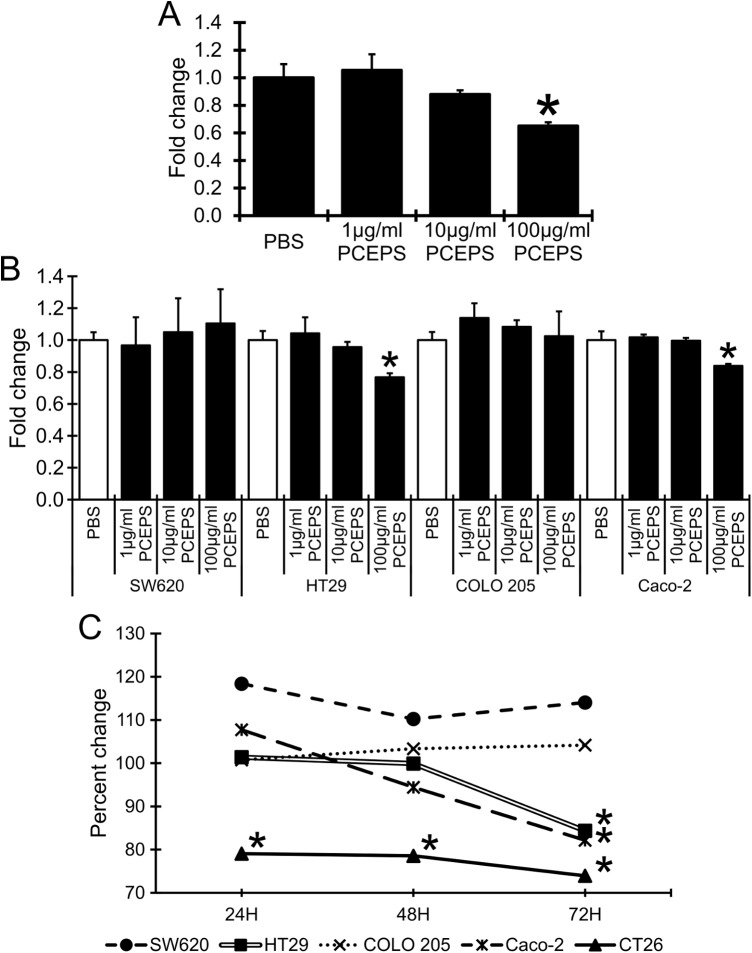
PCEPS dose- and time-dependently inhibited the growth of murine and human colon carcinoma cells in 2D cell culture. PCEPS treatment dose-dependently (1–100 μg/ml for 72 hrs, A-B) and time-dependently (24, 48 and 72 hrs treatment with 100 μg/ml PCEPS, C) attenuated the growth of CT26 murine colon carcinoma cells, HT29 and Caco-2, but not SW620 and COLO 205 human colon carcinoma cells. The cell growth was evaluated by MTT assay. Results are presented as mean ± SD (n = 3). *, P<0.05 compared to PBS-treated control by Tukey’s test or t-test.

**Fig 2:**
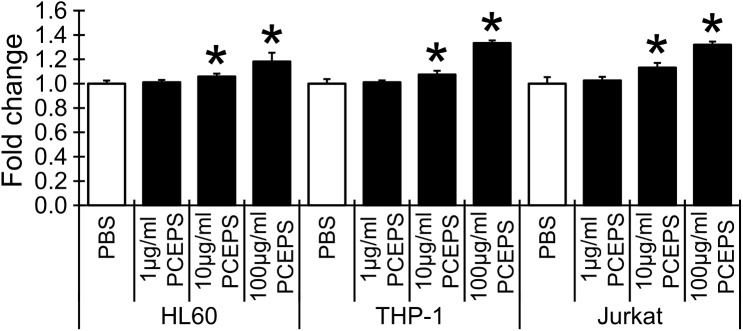
PCEPS dose-dependently stimulated the growth of immune cells. The dose-dependent effect of PCEPS (1–100 μg/ml for 48 hrs) on the growth of HL-60, THP-1 and Jurkat cells was evaluated by MTT assay. Results are presented as mean ± SD (n = 3). *, P<0.05 compared to PBS-treated control by Tukey’s test.

To clarify the effect of the water extract from the *Parachlorella kessleri* exopolysaccharide, PCEPS, on the growth of colon cancer cells, a MTT assay was conducted. First, the specificity of PCEPS’s effect on several colon cancer cells was evaluated. CT26 murine colon carcinoma cells, SW620, HT29, COLO 205 and Caco-2 human colon carcinoma cells were treated with PCEPS (1–100 μg/ml) for 72 hrs in 2D cell culture. The PCEPS treatment dose-dependently (Fig 1A and 1B) and time-dependently (Fig 1C) attenuated the growth of CT26, HT29 and Caco-2 colon carcinoma cells (P<0.05), but not SW620 and COLO 205 cells. The time-dependent growth inhibition curves clearly indicated that CT26 cell growth was most sensitively inhibited by the PCEPS treatment (Fig 1C). The growth of CT26 cells was decreased by treatments with 10 (12.1% decrease, n.s.) and 100 μg/ml PCEPS (35.0% decrease, P<0.05) at 72 hrs after the treatment (Fig 1A). Notably, the PCEPS treatment with 100 μg/ml inhibited the CT26 cell growth significantly at all three time points (24, 48 and 72 hrs), whereas the significant growth inhibition was detected at 72 hrs after the treatment in both HT29 and Caco-2 cells (Fig 1C). These results suggest that PCEPS treatment dose- and time-dependently attenuates the growth of multiple colon cancer cells. In contrast, PCEPS dose-dependently stimulated the growth of HL-60, THP-1 and Jurkat cells at 48 hrs after treatment (Fig 2). PCEPS treatment did not show any effect on mouse normal mesothelial cell growth at doses of 1–100 μg/ml for 24–72 hrs. These results may suggest that PCEPS contains immune cell activating molecules as well as cancer attenuating molecules which have negligible effect on normal cell growth.

### LPS treatment bidirectionally altered the growth of murine and human colon carcinoma cells, and slightly stimulated the growth of human immune cells

**Fig 3:**
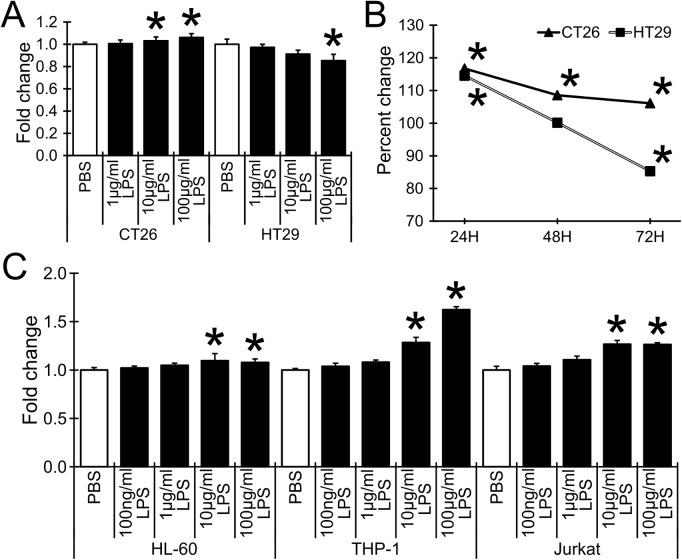
Effect of LPS on the growth of colon carcinoma cells and immune cells. (A-B) CT26 and HT29 colon carcinoma cells were treated with LPS dose-dependently (1–100 μg/ml for 72 hrs, A) and time-dependently (24, 48 and 72 hrs treatment with 100 μg/ml LPS, B). (C) Human immune cells (HL-60, THP-1 and Jurkat cells) were treated with LPS dose-dependently (1–100 μg/ml for 48 hrs). The cell growth was evaluated by MTT assay. Results are presented as mean ± SD (n = 5). *, P<0.05 compared to PBS-treated control by Tukey’s test or t-test.

As shown in Fig 3A, LPS treatment slightly but significantly increased the cell growth of CT26 murine colon carcinoma cells, but significantly attenuated the growth of HT29 human colon carcinoma cells. Time course study showed that LPS treatment increased CT26 cell growth even at 24 hrs after treatment and sustained the cell growth. On the contrary, HT29 cell growth was significantly higher than the control at 24 hrs after treatment, but gradually decreased to significantly low growth at the 72hrs time point (Fig 3B). The effect of LPS on cancer cell growth is clearly different from the effect of PCEPS (Figs 1 and 2). LPS treatment slightly but consistently stimulated the growth of all three types of immune cells (HL-60, THP-1 and Jurkat cells) in a dose-dependent manner (1–100 μg/ml for 48 hrs, Fig 3C). These results suggest that LPS functions to control the growth of colon carcinoma cells differently from PCEPS.

### PCEPS treatment stimulated the growth of splenocytes and bone marrow cells

**Fig 4:**
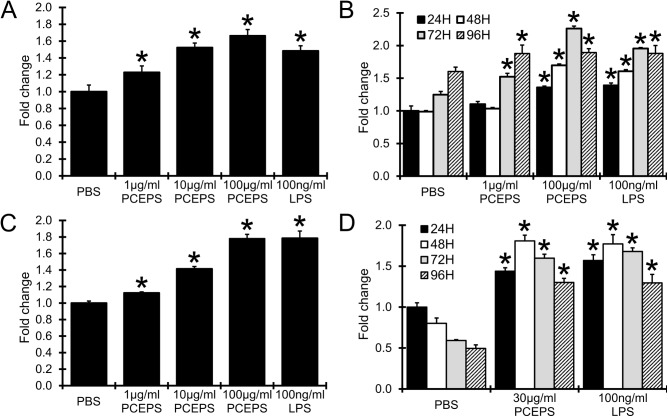
PCEPS dose- and time-dependently stimulated the growth of murine splenocytes and bone marrow cells in cell culture. PCEPS treatment dose-dependently (1–100 μg/ml for 48 hrs) and time-dependently (24, 48 72 and 96 hrs treatment with 1–100 μg/ml PCEPS) stimulated the growth of mouse SPLs (A and B) and BMCs (C and D). SPLs and BMCs were also treated with 100 ng/ml LPS as a control. The cell growth was evaluated by MTT assay. Results are presented as mean ± SD (n = 3). *, P<0.05 compared to PBS-treated control by Tukey’s test or t-test.

It has been reported that *Chlorella vulgaris* extract is capable of stimulating the growth of immune cells [10, 11, 13, 15, 16]. In the present study, therefore, the growth stimulation effect of PCEPS on immune cells was evaluated using SPLs and BMCs. PCEPS treatment (1–100 μg/ml) significantly increased the growth of both SPLs (Fig 4A and 4B) and BMCs (Fig 4C and 4D) in a dose- and time-dependent manner. This PCEPS-dependent growth stimulation effect was detected in lower concentrations at 1–100 μg/ml (Fig 4A and 4C). A positive control, LPS also exhibited similar results in both SPLs and BMCs.

### PCEPS-dependent growth stimulation of splenocytes was inhibited by TLR4 inhibitors

**Fig 5:**
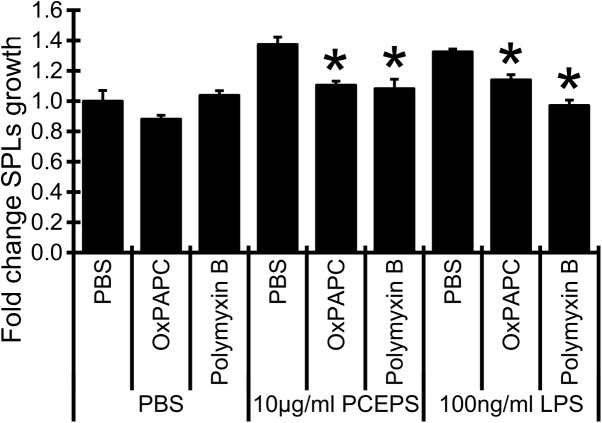
Evaluation of the TLRs involvement in the PCEPS-dependent growth stimulation of splenocytes in cell culture. SPLs were stimulated with 10 μg/ml PCEPS and 100 ng/ml LPS (TLR4 agonist) in the presence of 15 μg/ml OxPAC (TLR2 and TLR4 inhibitor) and 50 μg/ml Polymyxin B (an inhibitor of LPS-induced TLR4 activation). Inhibitory effect of TLR inhibitors on SPL growth was evaluated by MTT assay at 48 hrs after the treatment. *, P<0.05 compared to PBS-treated SPLs in each group by Tukey’s test.

It has been reported that water extracts from *C*. *vulgaris* [17] and mushroom polysaccharide Krestin [38] stimulate antitumor immune responses *via* TLR2. To evaluate the involvements of TLRs in PCEPS-dependent growth stimulation of SPLs, SPLs were treated with either 10 μg/ml PCEPS, or 100 ng/ml LPS (TLR4 agonist) in the presence/absence of 15μg/ml OxPAC (TLR2 and TLR4 inhibitor) and 50μg/ml Polymyxin B (TLR4 inhibitor). The results are summarized in Fig 5. The growth stimulatory effect by PCEPS was completely blunted by OxPAPC (19.5% decrease, P<0.05) and polymyxin B (21.2% decreased, P<0.05). In the parallel control study, treatment with the TLR4 agonist LPS significantly increased the growth of SPLs and its growth was inhibited by OxPAPC and polymyxin B (13.96% decrease (P<0.05) in OxPAPC and 26.75% decrease (P<0.05) in polymyxin B). These results suggest that both TLR2 and 4 are involved in PCEPS-dependent growth stimulation of splenocytes.

### PCEPS treatment increased CD8^+^ T cell population in bone marrow cells

**Fig 6:**
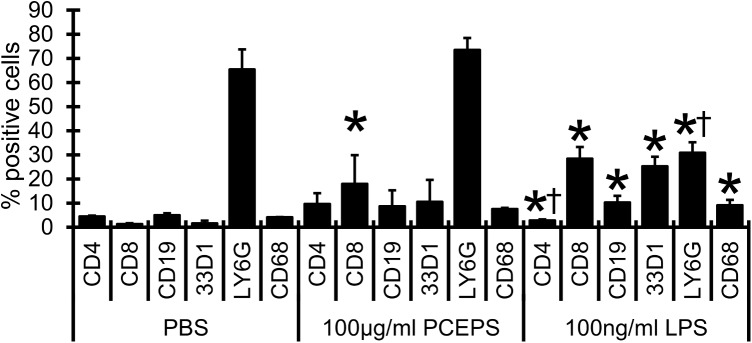
PCEPS treatment increased various immune cell populations in primary cultured mouse bone marrow cells. BMCs were collected from mouse hind legs and were seeded into 6-well plates and treated with 100 μg/ml PCEPS and 100 ng/ml LPS for 48 hrs. These BMCs were labeled with either anti-CD4, CD8b, CD19, 33D1, LY6G or CD68 antibodies and relative quantities were analyzed by flow cytometry. PBS and LPS were used as the negative and positive control, respectively. Results are presented as mean ± SD (n = 3). *, P<0.05 compared to PBS control group by t-test. †, P<0.05 compared to PCEPS-treated group by t-test.

PCEPS treatment stimulated the growth of BMCs (Fig 4C and 4D). Detailed analysis of the changes of immune cell populations in BMCs was performed using flow cytometry. As shown in Fig 6, treatment with 100 μg/ml PCEPS increased CD4^+^ (118.1% increase as compared with PBS control group), CD8^+^ (1,312.7% increase, P<0.05), CD19^+^ (74.9% increase), 33D1^+^ (563.9% increase), LY6G^+^ (12.4% increase) and CD68^+^ (81.0% increase) cells. Although all tested cell populations were increased by PCEPS treatment, increase in only CD8^+^ cell population was statistically significant. Increases of antigen presenting cell populations such as CD19^+^ (B cells), 33D1^+^ (DCs) and CD68^+^ (macrophages) cells were large, but their increases were not statistically significant due to large variations. In the LPS treatment group (100 ng/ml), CD8^+^ (2,132.7% increase as compared to PBS control group, P<0.05), CD19^+^ (105.8% increase, P<0.05), 33D1^+^ (1,494.4% increase, P<0.05) and CD68^+^ (120.9% increase, P<0.05) cells were increased. In contrast, CD4^+^ (37.2 and 71.2% decrease as compared to PBS control and PCEPS group, respectively, P<0.05) and LY6G^+^ (52.8 and 58.0% decrease, P<0.05) were significantly decreased. In the BMCs treated with LPS, antigen presenting cell population was notably increased compared with that of PCEPS-treated group. These results may suggest that PCEPS treatment increases cell populations related to the adaptive immune system.

### PCEPS treatment enhanced HL-60 and Jurkat cell antitumor activities in the spheroid growth of CT26 cells *in vitro*

**Fig 7:**
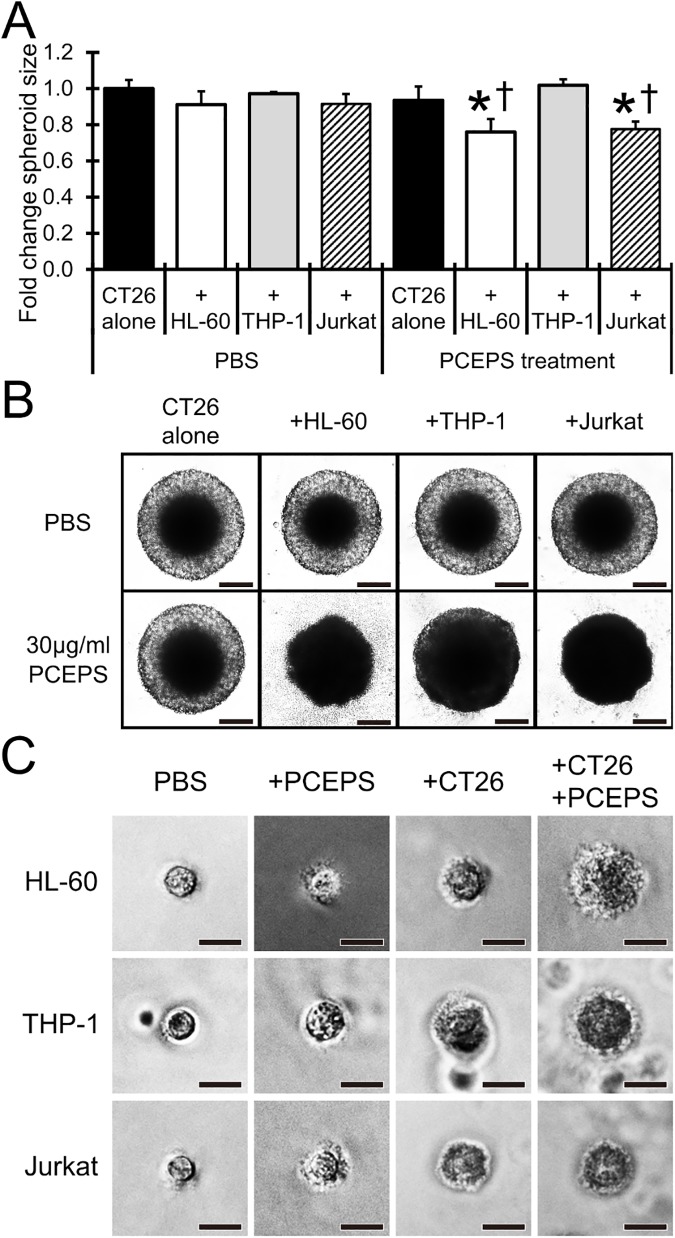
PCEPS treatment attenuated the spheroid growth of CT26 colon carcinoma cells in 3D cell culture in the presence of various immune cells. The CT26 cells were grown on a U-shaped agar matrix which contains HL-60, THP-1 or Jurkat cells (Day 0). The PCEPS (30 μg/ml) was treated twice at Day 1 and Day 4. The size of the spheroid was measured at Day 8 and Day 10 (A). The values are presented as fold change of spheroid size compared with Days 8 and 10 (Day10/Day 8). Results are presented as mean ± SD (n = 5). *, P<0.05 compared to PBS-treated CT26 cells alone group by Tukey’s test. †, P<0.05 compared to same group in PBS-treated group by t-test. (B) Typical pictures of spheroid in each group. Scale bar in each picture represents 100 μm. (C) Typical morphologies of HL-60, THP-1 and Jurkat cells in agar matrix of each treatment group. Scale bar in each picture represents 20 μm.

**Fig 8:**
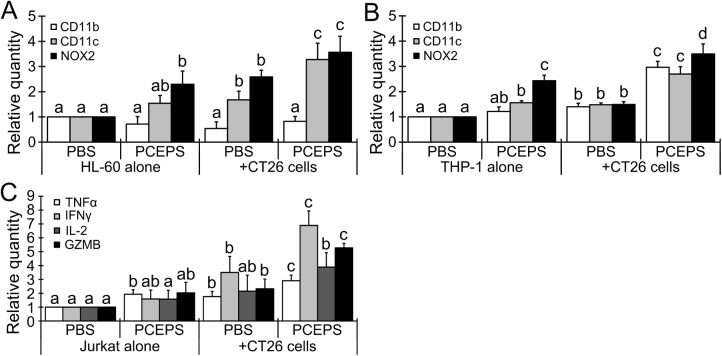
PCEPS treatment increased expression of differentiation marker and reactive oxygen-related genes in immune cells co-cultured with PCEPS-treated CT26 colon carcinoma cells in Transwell cell culture. The HL-60, THP-1 or Jurkat cells in the bottom well of 6-well plates and CT26 cells in the insert of Transwell were co-cultured (Day 0). The PCEPS (30 μg/ml) treatment was provided at Day 1, Day 3 and Day 5. Total RNA was collected from individual immune cells in the 6 well plate at Day 6. Gene expression in HL-60 (A), THP-1 (B) and Jurkat (C) cells were measured by RT-qPCR. Results are presented as mean ± SD (n = 4) of relative quantity. a-d, P<0.05 between different characters in each target gene by t-test.

The *in vitro* spheroid assay is an effective and a reproducible method to mimic a tumor microenvironment, therefore it is useful to evaluate the effect of therapeutics prior to an animal study [34]. As shown in Fig 7A, co-culture with HL-60, THP-1 and Jurkat cells did not attenuate the spheroid growth of CT26 cells in the PBS-treated group. Although the treatment with 30 μg/ml PCEPS alone also did not show any growth inhibition effect, the spheroid growth of CT26 cells was significantly attenuated with the combination of the PCEPS treatment with the co-culture of HL-60 (P<0.05 as compared with PCEPS alone and HL-60 cell alone co-cultured CT26 spheroids) or Jurkat cell (P<0.05 as compared with PCEPS alone and Jurkat cell alone co-cultured CT26 spheroids). Although the combination treatment with PCEPS and THP-1 cells significantly decreased the transparency in the peripheral area of the spheroid, this combination treatment did not decrease the spheroid’s size (Fig 7B). Morphological analysis of co-cultured immune cells in the spheroid assay revealed that PCEPS treatment alone or co-culturing with CT26 spheroids only slightly modified the morphologies of HL-60, THP-1 and Jurkat cells (Fig 7C). However, their morphologies were drastically changed by the combination treatment with PCEPS in the presence of a CT26 spheroid. These morphological analyses may suggest that PCEPS triggers immune cell differentiations and that the PCEPS effect is significantly enhanced in the presence of cancer cells. Further analysis of immune cells in the presence of PCEPS-treated CT26 cells was conducted by measuring gene expression using Transwell culture system (Fig 8). While only CD11c expression was significantly upregulated in HL-60 cells, both CD11b and CD11c expression were significantly upregulated in THP-1 cells co-cultured with PCEPS-treated CT26 cells. Analysis of genes related to the reactive oxygen species production and T cell activation revealed that significant upregulation of NOX2 in HL-60 and THP-1 cells, and TNFα, IFNγ and GZMB in Jurkat cells were observed as compared to PBS- or PCEPS alone-treated immune cells and immune cells co-cultured with PBS-treated CT26 cells. It is strongly suggested that PCEPS assists immature immune cells to differentiate into antitumor immune cells, thereby inhibiting CT26 cell tumor spheroid growth.

### Intraperitoneal injection of PCEPS attenuated the growth of CT26 murine colon carcinoma in a peritoneal dissemination model

**Fig 9:**
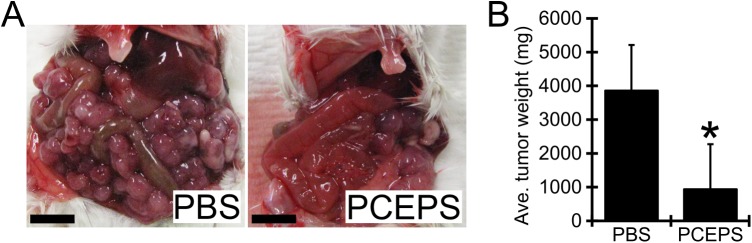
PCEPS treatment significantly attenuated the growth of CT26 cell tumors in mouse peritoneal cavity. The CT26 cells (2.5x10^5^) suspended in 200μl PBS was inoculated in peritoneal cavity of Balb/c mice. The PCEPS treatment (10 mg/kg, IP, every other day) was started one week after cancer cell inoculation and carried out 11 days (a total of 6 times injections). All mice were sacrificed three weeks after CT26 cell injection. (A) Macroscopic view of typical peritoneal cavity in PBS and PCEPS-treated mice. Scale bar in each picture represents 5 mm. (B) Average tumor weight in each treatment group was presented in the bar graphs. Results are presented as mean ± SD (n = 6). *, P<0.05 as compared with the tumor weight in PBS-treated group by t-test.

**Fig 10:**
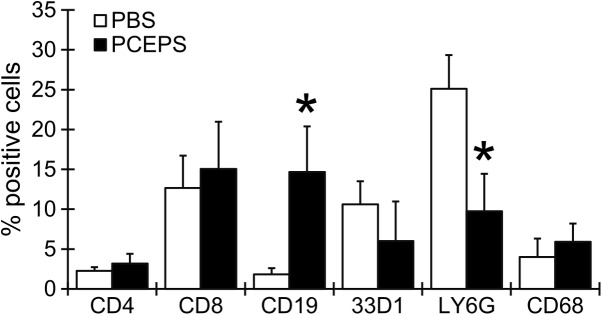
PCEPS treatment caused changes in leukocyte populations in ascites of CT26 cell tumor-bearing mice. The CT26 cells (2.5x10^5^) suspended in 200μl PBS was inoculated in the peritoneal cavity of Balb/c mice. The PCEPS treatment (10 mg/kg, IP, every other day) was started one week after cancer cell inoculation and continued for a total of 3 times. Ascites was collected two days after last treatment. Leukocytes in the ascites were labeled with anti-CD4, CD8b, CD19, 33D1, LY6G and CD68 antibodies and their populations were analyzed by flow cytometry. PBS was used as the negative control treatment. Results are presented as mean ± SD (n = 4). *, P<0.05 compared to PBS control group by t-test.

**Fig 11:**
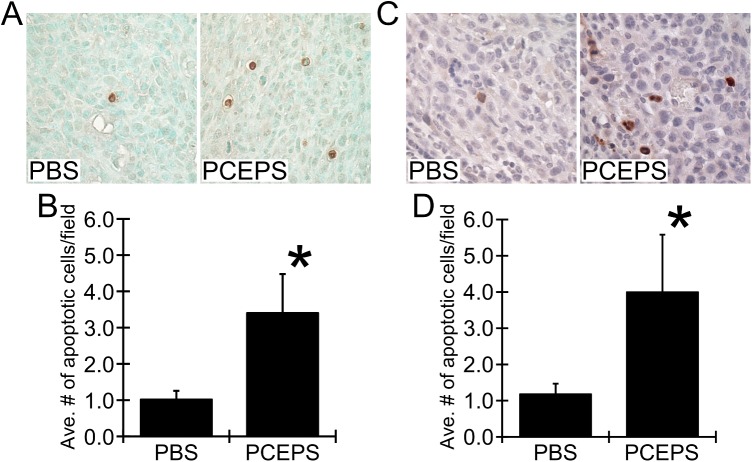
PCEPS treatment significantly increased the number of apoptotic cells in CT26 cell tumors in mouse peritoneal cavity. (A) Microscopic images of immunohistochemistry for TUNEL assay. (B) The TUNEL positive cells were significantly increased in the PCEPS-treated tumors. (C) Microscopic images of immunohistochemistry by anti-cleaved caspase-3. (D) The cleaved caspase-3 positive cells were also significantly increased in the PCEPS-treated tumors. Results are presented as mean ± SD (n = 5–6). *, P<0.05 as compared to the level of the PBS-treated control tumors by t-test.

To evaluate an antitumor effect of PCEPS *in vivo*, 10 mg/kg PCEPS was injected into the peritoneal cavity of the CT26 cell tumor-bearing mice (n = 6/group, 2.5x10^5^ cells). The treatment was started one week post tumor cell injection and was given every other day for 11 days. As shown in Fig 9A, the PBS control group developed a large number of 1–5 mm diameter tumor nodules in the entire greater omentum, whereas the PCEPS-treated group developed only a few visible tumor nodules. Average tumor weight (mg) in PCEPS-treated group (937.4±1,350.5, P<0.05) was significantly smaller than that of PBS treatment group (3,861.6±1,335.2) (Fig 9B). As shown in S1 and S2 Figs, PCEPS treatment did not influence organ weight in normal and tumor-bearing mice. Although the spleen weight increased in tumor-bearing mice compared with that of normal mice (S1 Fig), spleen weight increase is commonly observed in tumor bearing mice as tumors develop. Since PCEPS treatment induced increases of various leukocyte numbers in BMCs *in vitro* (Fig 6) and morphology changes in cultured immune cells (Fig 7), it suggests that PCEPS treatment may influence the host immune system. To clarify this hypothesis, immune cell populations in ascites collected from PBS- or PCEPS-treated tumor-bearing mice were investigated by flow cytometry. As shown in Fig 10, PCEPS treatment increased CD4^+^ (40.2% increase as compared to PBS control group), CD8^+^ T lymphocyte (18.9% increase), CD19^+^ B cell (700.3% increase, P<0.05) and CD68^+^ macrophage (47.8% increase) populations. In contrast, 33D1^+^ dendritic cell (43.5% decrease) and LY6G^+^ neutrophil (61.2% decrease) populations were decreased as compared to PBS-treated group. Significant increase of CD19^+^ B cell in PCEPS-treated mice suggest that PCEPS treatment may induce an adaptive immune response to the PCEPS. Immunohistochemical analysis of apoptotic cells in tumor nodules clearly suggested that the PCEPS treatment significantly increased apoptotic cells in PCEPS-treated CT26 cell tumors compared to PBS-treated tumors (Fig 11). These results clearly indicate that PCEPS treatment is effective in inhibition of colon carcinoma growth in mice.

## Discussion

Although chlorella and its extracts are food sources with high nutritional value, increasing publications indicate that chlorella extracts are novel sources for therapeutics of various diseases including cancers [3]. Most of the previous medical studies have used water extracts of *C*. *Vulgaris and C*. *Pyrenoidosa* among multiple chlorella species [4–18]. Nevertheless, *Parachlorella*, a genus of green algae that contains two species (*P*. *Kessleri* and *P*. *beijerinckil*) and belongs to chlorellales, has been limitedly used in medicinal fields. However, recent discoveries indicate that *Parachlorella kessleri* produces relatively large amounts of exopolysaccharides which coat the outside of their algae cell membrane [39]. Although these exopolysaccharides play a protective role against environmental stress and chemical toxins [21, 23], their functions in medicinal chemistry is yet to be clear. Accordingly, we have prepared a water extract from partially purified exopolysaccharides of *Parachlorella Kessleri*,termed PCEPS and evaluated their antitumor and immune modulatory abilities in *in vitro* and *in vivo*.

The effect of PCEPS on the growth of murine and human colon cancer cell lines was evaluated in both 2D and 3D cell cultures. PCEPS moderately, but significantly attenuated the growth of CT26 murine colon carcinoma cells in 2D cell culture (Fig 1A and 1C). This attenuating effect of PCEPS on the colon carcinoma was also observed in human cell lines, HT29 and Caco-2, but not in SW620 and COLO 205 (Fig 1B and 1C). Both HT29 and Caco-2 cell lines have an epithelial origin, whereas both SW620 and COLO 205 are established from colon carcinoma-derived metastatic tumors [40, 41]. These different origins of each cell line may describe the different responsiveness to PCEPS. In contrast, this growth inhibition effect was not detected in 3D spheroid culture (Fig 7). It is known that 3D culture condition more closely mimics natural tumor growth than that in 2D culture condition [34]. For this reason, 3D culture methods such as clonogenic assay [42–44] and spheroid assay [34] have been applied to the evaluation and screening of novel therapeutics for cancer treatments. Present study results, therefore, may suggest that the cytotoxic antitumor effect of PCEPS alone is not strong enough to inhibit 3D spheroid growth of CT26 cells within a short time. In addition, limitation of the MTT assay in monitoring cell proliferation should be considered since MTT assay is known to be influenced by metabolic status of cells [45].

In contrast, PCEPS treatment clearly attenuated spheroid growth of CT26 cells when they were co-cultured with HL-60 or Jurkat cells (Fig 7). These two cell lines are frequently used as typical immune cells alternative to mature immune cells collected from peripheral blood. For example, Jurkat cells were used for the evaluation of the IL-2-induced granzyme B production, which is released from cytotoxic T cells and natural killer cells and induces apoptosis in adjacent malignant cells [46, 47]. Likewise, HL-60 cells were used for the determination of neutrophil-specific antibacterial activity using neutrophil-like cells differentiated from HL-60 [48, 49]. Accordingly, it is thought that the co-culture of the cancer cells with the above mentioned immune cells conducted in the present study mimicked a tumor microenvironment closer to biological conditions than the single spheroid culture of cancer cells. Therefore, results suggest that the PCEPS-induced antitumor effect in the presence of immature immune cells, either neutrophilic promyeloblasts (HL-60 cells) or lymphoblasts (Jurkat cells) in 3D spheroid culture is attributable to the collaborative effect of both CT26 cells and immune cells. These results further suggest that PCEPS induces functional differentiations of these immature immune cells in collaboration with cancer cells. Indeed, morphologies of these immature cells have drastically enlarged and became dendritic cell-type structure in the spheroid culture (Fig 7C). This PCEPS induced functional differentiations of these immune cells co-cultured with cancer cells was also confirmed by the changes of neutrophil, monocyte/macrophage or T cell activation-associated mRNA level (Fig 8). This PCEPS-induced local differentiation/activation of two types of immune cells is a potentially useful mechanism in targeting cancer therapy applicable to both primary and metastatic cancer. To the best of our knowledge, this is the first study to report that PCEPS stimulates differentiations of two types of immature immune cells thereby, inducing cell death of cancer cells in co-culture.

The finding of immune stimulatory effect of PCEPS in the 3D spheroid assay was also supported from a different angle. Cell culture studies with primary cultured mouse SPLs and BMCs indicated that PCEPS stimulates the growth of both cell types *via* either TLR2 or TLR4 signaling (Figs 4 and 5). Furthermore, flow cytometry analysis revealed that PCEPS treatment increased various leucocytes including antigen presenting cells and T lymphocyte, particularly CD8^+^ T cell population in BMCs (Fig 6). These results, along with the 3D tumor spheroid assay, suggest that the PCEPS treatment may stimulate both growth and differentiation of multiple leucocyte populations *via* either TLR2 or TLR4 signaling. It is reported that LPS-like molecules are found in *chlorella sp*. (strain NC64A) [31, 32]. It appears that cell membranes of green algae contain lipopolysaccharides similar to those of LPS [31, 32]. Although the PCEPS preparation used for this study was found to contain a very low level of LPS or LPS-like molecules (0.1 ng per 1 μg PCEPS), the effects of LPS on the growth of colon carcinoma cells and immune cells were evaluated (Figs 1–3) and compared with those by the PCEPS. As shown in the Fig 3, high concentrations of LPS (100 μg/ml) stimulated the growth of CT26 cells and all three types of immune cells. Although extents of the growth stimulation of immune cells by PCEPS were higher than those by LPS, both molecules stimulated immune cell growth. However, PCEPS and LPS showed opposite reactions against CT26 cells (PCEPS decreased (Fig 1), but LPS significantly increased cell growth (Fig 3)). In addition, bone marrow cell responses to PCEPS/LPS (Fig 6) indicate that 33D1 positive dendritic cells responded to only LPS. It is apparent that PCEPS is functionally different from LPS in CT26 cell/dendritic cell growth stimulations. These results clearly indicate that biological activities of PCEPS are different from those of LPS and LPS-like molecule’s contribution to PCEPS’s activities appears to be negligible. However, to further clarify the biological activities of PCEPS, determination of the structural identify of the active compound(s) in PCEPS is essential.

The solid inhibitory ability against colon carcinoma cells in both 2D and 3D cell culture studies and the robust growth and differentiation stimulatory actions in various leucocytes with negligible cytotoxicity compelled *in vivo* efficacy of PCEPS. In the present mouse study, a relatively small amount of PCEPS (10 mg/kg dissolved in PBS) was injected intraperitoneally every other day starting one week after cancer cell inoculation. As shown in Figs 9 and 11, this PCEPS treatment significantly attenuated the growth of murine colon carcinoma cells by inducing apoptosis in tumor cells as compared to the PBS-treated mice in a peritoneal dissemination model. Taken together, these results suggest that PCEPS effectively inhibited the growth of colon carcinoma cells directly and indirectly through immune cell activations in a tumor microenvironment, thereby significantly attenuating colon tumor growth in mice. The major antitumor mechanism by which PCEPS inhibited tumor growth in mice appears to be the stimulation of antitumor immune function since 3D tumor spheroid growth was significantly inhibited only in the presence of neutrophilic promyeloblasts, HL-60, and lymphoblasts, Jurkat cells. This speculation is supported by the PCEPS-dependent increases of various adaptive immune cells in ascites (Fig 10) and upregulations of reactive oxygen species-related genes in HL-60, and T cell cytotoxicity-related cytokines, such as TNFα, IFNγ and GZMB in Jurkat cells (Fig 8). However, a detailed mechanism that inhibits tumor growth *in vivo* must be clarified by another study.

In conclusion, our novel *Parachlorella kessleri* extract, PCEPS, can moderately inhibit murine colon carcinoma cells, but significantly stimulate the growth of primary cultured mouse SPLs and BMCs. PCEPS treatment also significantly increased CD8^+^ T cell population in primary cultured mouse BMCs. In a 3D spheroid culture, PCEPS treatment significantly attenuated the spheroid growth of murine colon carcinoma cells in the presence of neutrophilic promyeloblasts, HL-60, or lymphoblasts, Jurkat cells. In a murine colon carcinoma peritoneal dissemination model with syngeneic mice, PCEPS treatment markedly attenuated tumor growth. Although, further studies are required to confirm the *in vivo* safety of PCEPS by formal pharmacokinetics, pharmacodynamics, and multispecies toxicity studies, these data show that PCEPS could be a useful bioactive agent that can stimulate antitumor immune cells.

## Supporting information

S1 Fig Average organ weights in PBS- or PCEPS-treated normal or CT26 cell tumor-bearing mice at full scale PCEPS treatment (total 6 time treatments). Omenta (only normal mice), pancreases (only normal mice), lungs, livers, spleens and intestines in normal or tumor-bearing mice in each treatment group was dissected and weighed. Results are presented as mean ± SD (n = 6). *, P<0.05 as compared to the PBS-treated group by t-test.
(TIF)

S2 Fig Average tumor and organ weights in PBS- or PCEPS-treated normal or CT26 cell tumor-bearing mice at midpoint of the PCEPS treatment (total 3 time treatments). Tumors, lungs, livers, spleens and intestines in normal or tumor-bearing mice in each treatment group was dissected and weighed. Results are presented as mean ± SD (n = 4). *, P<0.05 as compared to the PBS-treated group by t-test.
(TIF)

## References

[1] Siegel RL, Miller KD, Jemal A. Cancer statistics, 2016. CA Cancer J Clin. 2016;66(1):7–30. Epub 2016/01/09. 10.3322/caac.21332 26742998

[2] American Cancer Society. Colorectal Cancer Facts & Figures 2014–2016. Atlanta: American Cancer Society 2014.

[3] Buono S, Langellotti AL, Martello A, Rinna F, Fogliano V. Functional ingredients from microalgae. Food Funct. 2014;5(8):1669–85. 10.1039/c4fo00125g 24957182

[4] Merchant RE, Andre CA, Sica DA. Nutritional supplementation with Chlorella pyrenoidosa for mild to moderate hypertension. J Med Food. 2002;5(3):141–52. Epub 2002/12/24. 10.1089/10966200260398170 12495586

[5] Okamoto K, Iizuka Y, Murakami T, Miyake H, Suzuki T. Effects of chlorella alkali extract on blood pressure in SHR. Jpn Heart J. 1978;19(4):622–3. Epub 1978/07/01. 731905 10.1536/ihj.19.622

[6] Sano T, Tanaka Y. Effect of dried, powdered Chlorella vulgaris on experimental atherosclerosis and alimentary hypercholesterolemia in cholesterol-fed rabbits. Artery. 1987;14(2):76–84. Epub 1987/01/01. 3566534

[7] Sano T, Kumamoto Y, Kamiya N, Okuda M, Tanaka Y. Effect of lipophilic extract of Chlorella vulgaris on alimentary hyperlipidemia in cholesterol-fed rats. Artery. 1988;15(4):217–24. Epub 1988/01/01. 3136759

[8] Ibusuki K, Minamishima Y. Effect of Chlorella vulgaris extracts on murine cytomegalovirus infections. Nat Immun Cell Growth Regul. 1990;9(2):121–8. Epub 1990/01/01. 1693753

[9] Hasegawa T, Okuda M, Makino M, Hiromatsu K, Nomoto K, Yoshikai Y. Hot water extracts of Chlorella vulgaris reduce opportunistic infection with Listeria monocytogenes in C57BL/6 mice infected with LP-BM5 murine leukemia viruses. Int J Immunopharmacol. 1995;17(6):505–12. Epub 1995/06/01. 7499027 10.1016/0192-0561(95)00035-z

[10] Tanaka K, Konishi F, Himeno K, Taniguchi K, Nomoto K. Augmentation of antitumor resistance by a strain of unicellular green algae, Chlorella vulgaris. Cancer Immunol Immunother. 1984;17(2):90–4. Epub 1984/01/01. 6565519 10.1007/BF00200042 PMC11039187

[11] Konishi F, Tanaka K, Himeno K, Taniguchi K, Nomoto K. Antitumor effect induced by a hot water extract of Chlorella vulgaris (CE): resistance to Meth-A tumor growth mediated by CE-induced polymorphonuclear leukocytes. Cancer Immunol Immunother. 1985;19(2):73–8. Epub 1985/01/01. 3845850 10.1007/BF00199712 PMC11039246

[12] Miyazawa Y, Murayama T, Ooya N, Wang LF, Tung YC, Yamaguchi N. Immunomodulation by a unicellular green algae (Chlorella pyrenoidosa) in tumor-bearing mice. J Ethnopharmacol. 1988;24(2–3):135–46. Epub 1988/12/01. 3253484 10.1016/0378-8741(88)90145-6

[13] Tanaka K, Tomita Y, Tsuruta M, Konishi F, Okuda M, Himeno K, et al Oral administration of Chlorella vulgaris augments concomitant antitumor immunity. Immunopharmacol Immunotoxicol. 1990;12(2):277–91. Epub 1990/01/01. 10.3109/08923979009019673 2229925

[14] Noda K, Ohno N, Tanaka K, Kamiya N, Okuda M, Yadomae T, et al A water-soluble antitumor glycoprotein from Chlorella vulgaris. Planta Med. 1996;62(5):423–6. Epub 1996/10/01. 10.1055/s-2006-957931 8923807

[15] Tanaka K, Yamada A, Noda K, Hasegawa T, Okuda M, Shoyama Y, et al A novel glycoprotein obtained from Chlorella vulgaris strain CK22 shows antimetastatic immunopotentiation. Cancer Immunol Immunother. 1998;45(6):313–20. Epub 1998/03/07. 9490201 10.1007/s002620050448 PMC11037613

[16] Justo GZ, Silva MR, Queiroz ML. Effects of the green algae Chlorella vulgaris on the response of the host hematopoietic system to intraperitoneal ehrlich ascites tumor transplantation in mice. Immunopharmacol Immunotoxicol. 2001;23(1):119–32. Epub 2001/04/27. 10.1081/IPH-100102573 11322644

[17] Hasegawa T, Matsuguchi T, Noda K, Tanaka K, Kumamoto S, Shoyama Y, et al Toll-like receptor 2 is at least partly involved in the antitumor activity of glycoprotein from Chlorella vulgaris. Int Immunopharmacol. 2002;2(4):579–89. 11962736 10.1016/s1567-5769(02)00002-4

[18] Ramos AL, Torello CO, Queiroz ML. Chlorella vulgaris modulates immunomyelopoietic activity and enhances the resistance of tumor-bearing mice. Nutr Cancer. 2010;62(8):1170–80. 10.1080/01635581.2010.513801 21058206

[19] Raposo MF, de Morais RM, Bernardo de Morais AM. Bioactivity and applications of sulphated polysaccharides from marine microalgae. Mar Drugs. 2013;11(1):233–52. Epub 2013/01/25. PubMed Central PMCID: PMC3564169. 10.3390/md11010233 23344113 PMC3564169

[20] Donot F, Fontana A, Baccou JC, Schorr-Galindo S. Microbial exopolysaccharides: Main examples of synthesis, excretion, genetics and extraction. Carbohyd Polym. 2012;87(2):951–62.

[21] El-Sheekh MM, Khairy HM, El-Shenody R. Algal production of extra and intra-cellular polysaccharides as an adaptive response to the toxin crude extract of Microcystis aeruginosa. Iranian J Environ Health Sci Eng. 2012;9(1):10 Epub 2013/02/02. PubMed Central PMCID: PMC3561052. 10.1186/1735-2746-9-10 23369164 PMC3561052

[22] Li P, Harding SE, Liu Z. Cyanobacterial exopolysaccharides: their nature and potential biotechnological applications. Biotechnology & genetic engineering reviews. 2001;18:375–404. 11530697 10.1080/02648725.2001.10648020

[23] Mohamed ZA. Polysaccharides as a protective response against microcystin-induced oxidative stress in Chlorella vulgaris and Scenedesmus quadricauda and their possible significance in the aquatic ecosystem. Ecotoxicology. 2008;17(6):504–16. Epub 2008/04/05. 10.1007/s10646-008-0204-2 18389369

[24] Ou Y, Xu S, Zhu D, Yang X. Molecular mechanisms of exopolysaccharide from Aphanothece halaphytica (EPSAH) induced apoptosis in HeLa cells. PLoS One. 2014;9(1):e87223 Epub 2014/01/28. PubMed Central PMCID: PMC3900761. 10.1371/journal.pone.0087223 24466342 PMC3900761

[25] Zheng W, Chen C, Cheng Q, Wang Y, Chu C. Oral administration of exopolysaccharide from Aphanothece halophytica (Chroococcales) significantly inhibits influenza virus (H1N1)-induced pneumonia in mice. Int Immunopharmacol. 2006;6(7):1093–9. Epub 2006/05/23. 10.1016/j.intimp.2006.01.020 16714212

[26] PANAC CO. LTD. Novel polysaccharide. Japan patent. 2014;JP2014-025035A.

[27] Doi C, Egashira N, Kawabata A, Maurya DK, Ohta N, Uppalapati D, et al Angiotensin II type 2 receptor signaling significantly attenuates growth of murine pancreatic carcinoma grafts in syngeneic mice. BMC Cancer. 2010;10:67 PubMed Central PMCID: PMC2846883. 10.1186/1471-2407-10-67 20181281 PMC2846883

[28] Bot J, Whitaker D, Vivian J, Lake R, Yao V, McCauley R. Culturing mouse peritoneal mesothelial cells. Pathology, research and practice. 2003;199(5):341–4. 10.1078/0344-0338-00427 12908525

[29] Mikheyskaya LV, Ovodova RG, Ovodov YS. Isolation and characterization of lipopolysaccharides from cell walls of blue-green algae of the genus Phormidium. Journal of bacteriology. 1977;130(1):1–3. PubMed Central PMCID: PMC235166. 404280 10.1128/jb.130.1.1-3.1977 PMC235166

[30] Osborn MJ, Rosen SM, Rothfield L, Zeleznick LD, Horecker BL. Lipopolysaccharide of the Gram-Negative Cell Wall. Science. 1964;145(3634):783–9. 14163315 10.1126/science.145.3634.783

[31] Armstrong MT, Theg SM, Braun N, Wainwright N, Pardy RL, Armstrong PB. Histochemical evidence for lipid A (endotoxin) in eukaryote chloroplasts. FASEB journal: official publication of the Federation of American Societies for Experimental Biology. 2006;20(12):2145–6. 16935939 10.1096/fj.05-5484fje

[32] Armstrong PB, Armstrong MT, Pardy RL, Child A, Wainwright N. Immunohistochemical demonstration of a lipopolysaccharide in the cell wall of a eukaryote, the green alga, Chlorella. The Biological bulletin. 2002;203(2):203–4. 10.2307/1543397 12414578

[33] Royce CL, Pardy RL. Endotoxin-like properties of an extract from a symbiotic, eukaryotic Chlorella-like green alga. J Endotoxin Res. 1996;3(6):437–44.

[34] Friedrich J, Seidel C, Ebner R, Kunz-Schughart LA. Spheroid-based drug screen: considerations and practical approach. Nat Protoc. 2009;4(3):309–24. 10.1038/nprot.2008.226 19214182

[35] Schmittgen TD, Livak KJ. Analyzing real-time PCR data by the comparative C(T) method. Nat Protoc. 2008;3(6):1101–8. 18546601 10.1038/nprot.2008.73

[36] Matsuzuka T, Rachakatla RS, Doi C, Maurya DK, Ohta N, Kawabata A, et al Human umbilical cord matrix-derived stem cells expressing interferon-beta gene significantly attenuate bronchioloalveolar carcinoma xenografts in SCID mice. Lung Cancer. 2010;70(1):28–36. Epub 2010/02/09. PubMed Central PMCID: PMC2930041. 10.1016/j.lungcan.2010.01.003 20138387 PMC2930041

[37] Ishiguro S, Cai S, Uppalapati D, Turner K, Zhang T, Forrest WC, et al Intratracheal Administration of Hyaluronan-Cisplatin Conjugate Nanoparticles Significantly Attenuates Lung Cancer Growth in Mice. Pharmaceutical research. 2016;33(10):2517–29. PubMed Central PMCID: PMC5007205. 10.1007/s11095-016-1976-3 27335023 PMC5007205

[38] Lu H, Yang Y, Gad E, Wenner CA, Chang A, Larson ER, et al Polysaccharide krestin is a novel TLR2 agonist that mediates inhibition of tumor growth via stimulation of CD8 T cells and NK cells. Clin Cancer Res. 2011;17(1):67–76. Epub 2010/11/12. PubMed Central PMCID: PMC3017241. 10.1158/1078-0432.CCR-10-1763 21068144 PMC3017241

[39] Coragliotti A. FS, Day AG., Decker SM., inventor; Solazyme, Inc., assignee. Microalgal polysaccharide compositions. United States patent US 20120202768. 2012 Aug 9.

[40] Semple TU, Quinn LA, Woods LK, Moore GE. Tumor and lymphoid cell lines from a patient with carcinoma of the colon for a cytotoxicity model. Cancer Res. 1978;38(5):1345–55. Epub 1978/05/01. 565251

[41] Leibovitz A, Stinson JC, McCombs WB 3rd, McCoy CE, Mazur KC, Mabry ND. Classification of human colorectal adenocarcinoma cell lines. Cancer Res. 1976;36(12):4562–9. Epub 1976/12/11. 1000501

[42] Kawabata A, Matsuzuka T, Doi C, Seiler G, Reischman J, Pickel L, et al C1B domain peptide of protein kinase Cgamma significantly suppresses growth of human colon cancer cells in vitro and in an in vivo mouse xenograft model through induction of cell cycle arrest and apoptosis. Cancer Biol Ther. 2012;13(10):880–9. Epub 2012/07/13. PubMed Central PMCID: PMC3414411. 10.4161/cbt.20840 22785210 PMC3414411

[43] Ishiguro S, Yoshimura K, Tsunedomi R, Oka M, Takao S, Inui M, et al Involvement of angiotensin II type 2 receptor (AT2R) signaling in human pancreatic ductal adenocarcinoma (PDAC): a novel AT2R agonist effectively attenuates growth of PDAC grafts in mice. Cancer Biol Ther. 2015;16(2):307–16. Epub 2015/03/11. PubMed Central PMCID: PMC4623015. 10.1080/15384047.2014.1002357 25756513 PMC4623015

[44] Ohta N, Ishiguro S, Kawabata A, Uppalapati D, Pyle M, Troyer D, et al Human umbilical cord matrix mesenchymal stem cells suppress the growth of breast cancer by expression of tumor suppressor genes. PLoS One. 2015;10(5):e0123756 Epub 2015/05/06. PubMed Central PMCID: PMC4420498. 10.1371/journal.pone.0123756 25942583 PMC4420498

[45] Sylvester PW. Optimization of the tetrazolium dye (MTT) colorimetric assay for cellular growth and viability. Methods in molecular biology. 2011;716:157–68. 10.1007/978-1-61779-012-6_9 21318905

[46] Huang C, Bi E, Hu Y, Deng W, Tian Z, Dong C, et al A novel NF-kappaB binding site controls human granzyme B gene transcription. J Immunol. 2006;176(7):4173–81. Epub 2006/03/21. 16547254 10.4049/jimmunol.176.7.4173

[47] Voskoboinik I, Whisstock JC, Trapani JA. Perforin and granzymes: function, dysfunction and human pathology. Nat Rev Immunol. 2015;15(6):388–400. Epub 2015/05/23. 10.1038/nri3839 25998963

[48] Brinkmann V, Reichard U, Goosmann C, Fauler B, Uhlemann Y, Weiss DS, et al Neutrophil extracellular traps kill bacteria. Science. 2004;303(5663):1532–5. Epub 2004/03/06. 10.1126/science.1092385 15001782

[49] Kawakami T, He J, Morita H, Yokoyama K, Kaji H, Tanaka C, et al Rab27a is essential for the formation of neutrophil extracellular traps (NETs) in neutrophil-like differentiated HL60 cells. PLoS One. 2014;9(1):e84704 Epub 2014/01/10. PubMed Central PMCID: PMC3880328. 10.1371/journal.pone.0084704 24404184 PMC3880328

