# Supplementary figures and images for: Exopolysaccharides extracted from Parachlorella kessleri inhibit colon carcinoma growth in mice via stimulation of host antitumor immune responses
Source: PLoS One. 2017 Apr 5;12(4):e0175064. doi: 10.1371/journal.pone.0175064 (PMC5381895; doi:10.1371/journal.pone.0175064)

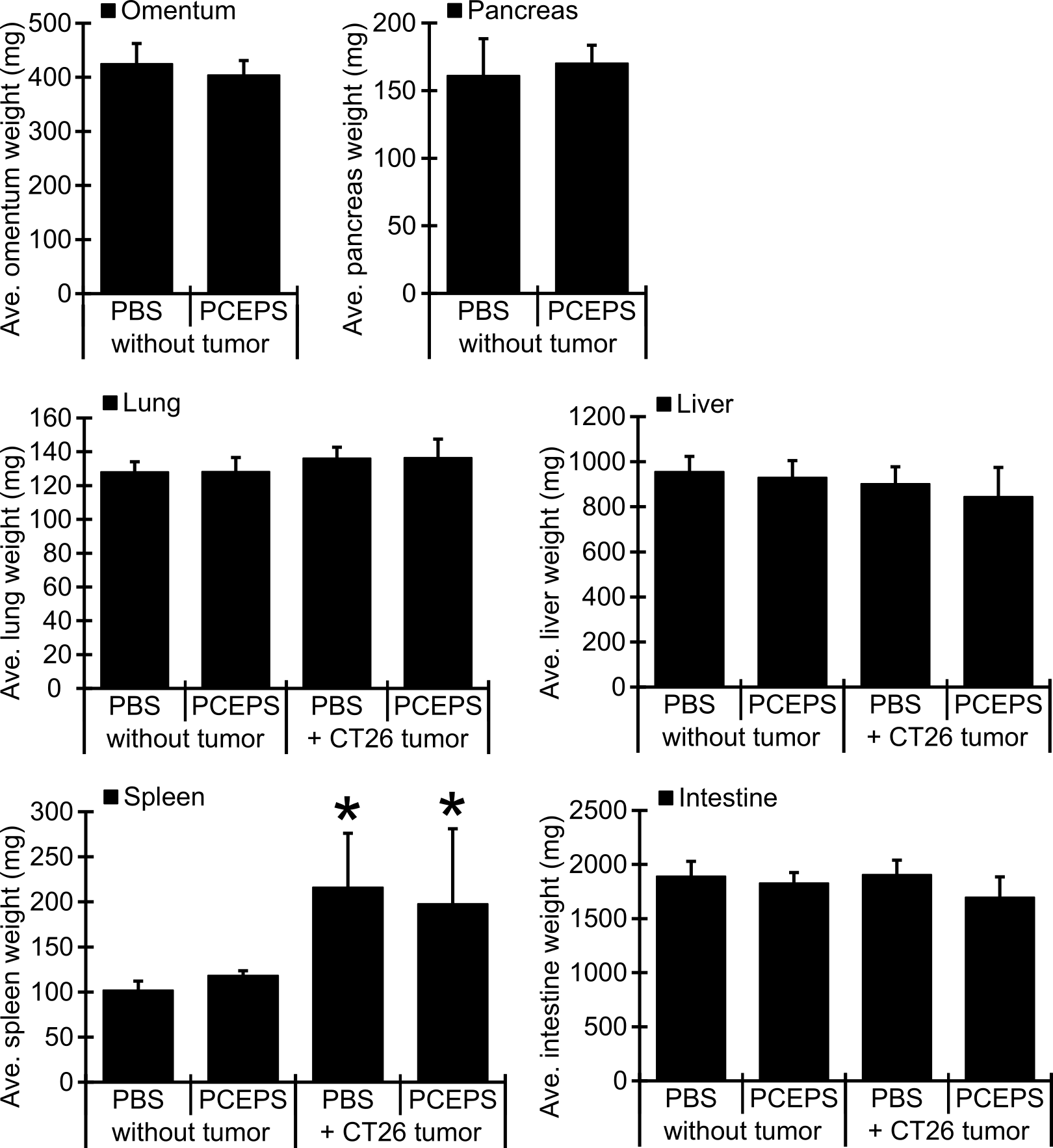

Supplement: S1 Fig — Omenta (only normal mice), pancreases (only normal mice), lungs, livers, spleens and intestines in normal or tumor-bearing mice in each treatment group was dissected and weighed. Results are presented as mean ± SD (n = 6). *, P<0.05 as compared to the PBS-treated group by t-test. (TIF) [file pone.0175064.s001.tif]

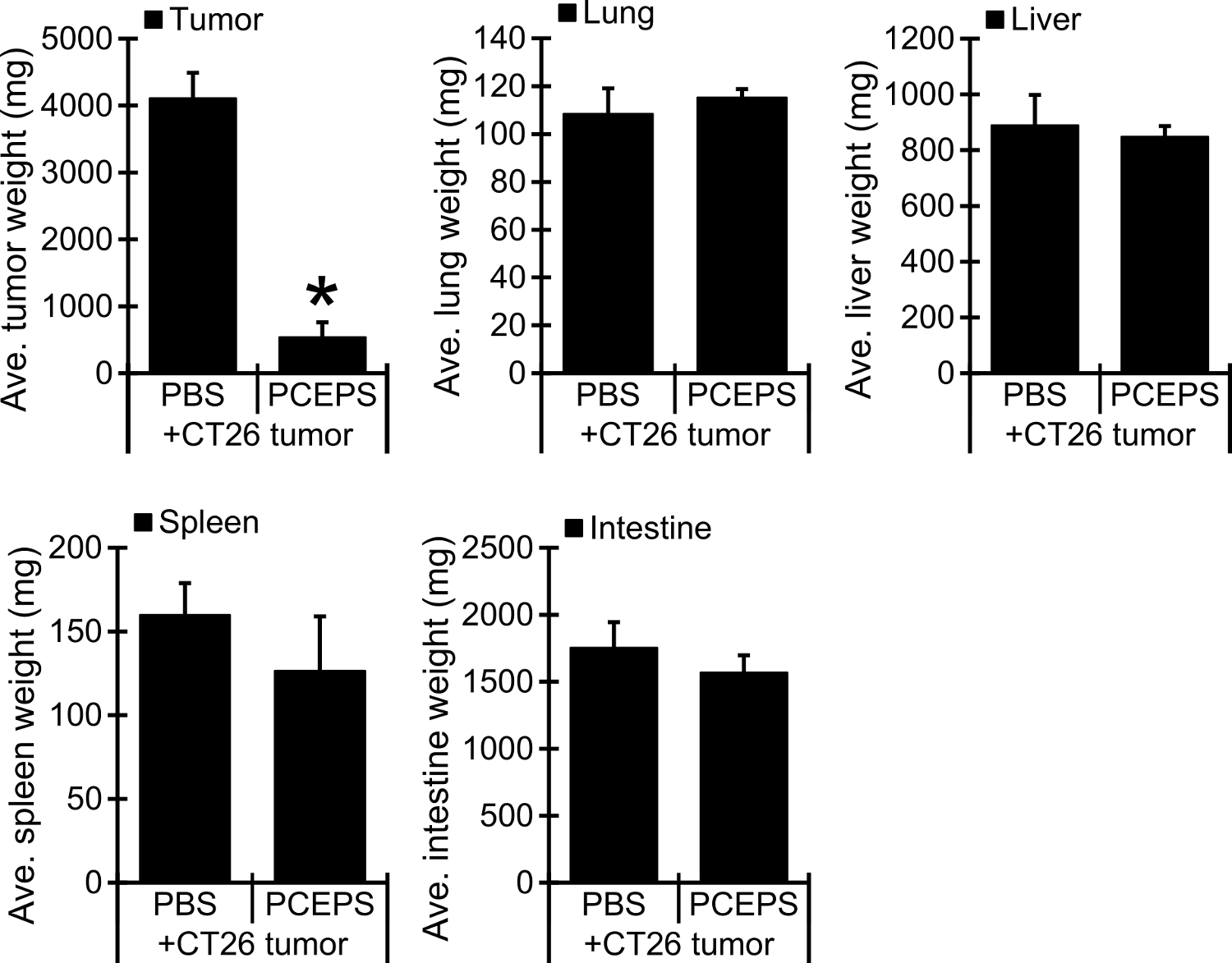

Supplement: S2 Fig — Tumors, lungs, livers, spleens and intestines in normal or tumor-bearing mice in each treatment group was dissected and weighed. Results are presented as mean ± SD (n = 4). *, P<0.05 as compared to the PBS-treated group by t-test. (TIF) [file pone.0175064.s002.tif]
